# Assessment of serum amylase, lipase and associated factors among patients with visceral leishmaniasis treated with sodium stibogluconate/paromomycin at University of Gondar Comprehensive Specialized Hospital, Northwest Ethiopia

**DOI:** 10.1371/journal.pone.0257229

**Published:** 2021-10-01

**Authors:** Tiget Ayelgn Mengstie, Hiwot Tezera Endale, Tadele Mulaw, Aman Mossa Abdella, Rezika Mohammed, Tabarak Malik, Gashaw Dessie

**Affiliations:** 1 Department of Biochemistry, School of Medicine, College of Medicine and Health Sciences, University of Gondar, Gondar, Ethiopia; 2 Leishimaniasis Research and Treatment Center, School of Medicine, University of Gondar, Gondar, Ethiopia; Babol University of Medical Science, ISLAMIC REPUBLIC OF IRAN

## Abstract

**Background:**

Visceral leishmaniasis (VL) is a life-threatening parasitic disease next to malaria, which is responsible for the death of 50,000 patients annually. It has three major clinical stages, including visceral, cutaneous, and mucocutaneous leishmaniasis. Ethiopia is one of the east African countries commonly affected with leishmanisis disease. There are many drugs for leishmaniasis, including sodium stibogluconate and paromomycin combined therapy. However, the adverse effect of those combined drugs is not well-defined. Therefore, the purpose of this study was to assess serum amylase, lipase, and associated factors among patients with VL treatment with those combined drugs.

**Methods:**

Hospital-based cross-sectional study was conducted at the University of Gondar Comprehensive Specialized Hospital Leishmaniasis Research and Treatment Center from February to September 2020 G.C. Simple random sampling technique was utilized to select study participants. The study participants who fulfill the inclusion criteria were included in the study with written informed consent. 5 ml of blood was withdrawn by an experienced health professional to analyze serum amylase and lipase level. Descriptive data was presented by tables, charts and graphs. Data was cleared, entered by Epi-data version 3.1 then transfer to STATA 14.1 SE version and for analysis paired t-test was used, for factors correlation and regression was used. Those factor variable who have p-value <0.25 was filtered and goes to multivariate regression and p-value <0.05 was considered as significant variables.

**Results:**

The result of this study showed that there was a significant mean difference between serum pancreatic amylase and lipase before and after treatment. The mean ± SD level of serum amylase after treatment showed a statistically significant elevation (P<0.001) as compared to its level before treatment. Similarly, the mean ± SD level of serum lipase after treatment showed a statistically significant elevation (P<0.001) as compared to its level before treatment. There was also significant association between age and baseline serum amylase as compared to serum amylase after treatment. Similarly, there was also significant relation of age and serum lipase with serum lipase after treatment.

**Conclusion:**

In this study, the level of serum amylase and lipase at treatment of cure was higher and there was an increase in mean serum amylase and lipase after a patient taking sodium stibogluconate and paromomycin combined drugs. Consequently, the elevated result of these biochemical profiles mainly associated with drug induced adverse effect and associated risk factors in VL patients.

## Introduction

Visceral leishimaniasis (VL) is a life-threatening parasitic disease caused by the Leishmania *donovani Complex* transmitted through a female vector phlebotomus, which is mostly found in warm tropical regions [1]. It is the largest global parasitic disease, responsible for about 50,000 deaths annually. The pathogenesis of VL involves the dissemination of the Leishmania parasite to different organs, presents with persistent fever, enlarged spleen, and blood abnormalities. Symptomatic VL is almost always fatal if it is untreated [2]. It has three major recognized forms of the disease, such as visceral, mucosal, and cutaneous. Visceral leishmaniasis(VL) is widely affected the population than the otherform of diseases [3]. More than 90% of all VL cases are disseminated in different countries, including India, Bangladesh, Nepal, Brazil, and Sudan [4]. Next to India, the second largest VL focus occurred in East African countries (Ethiopia, South Sudan, and Sudan), which accounts up to 30,000–40,000 new cases per year [5]. In Ethiopia, an estimated 2500 to 4000 new cases occur annually and over 3.2 million people from the total are at risk of acquiring infection [5]. The southern lowland area of Southern Nations, Nationalities and Peoples’ Regional State (SNNPR), including south-western savannah, south-eastern, the Woyto, and Segen River valleys are mostly affected by the disease In addition, northern lowland areas including, Metema and Humera are known VL foci in Ethiopia [6, 7]. Additionally, the prevalent and newly infected VL cases elevated in lower Omo plains of South West part of Ethiopia [8]. According to WHO bidirectional report held in Addis Ababa in 2015, the incidence of co-infection of leshimaniasis and HIV is expected to estimated within the range of 15–30%, which showed the extra complication of leshimaniasis [9]. The prevalence of VL is mainly correlated with worker displacement to highly endemic area and exposure of immune compromized individual to the pathogenic parasite. In addition to this, the risk of VL is elevated in the area with inadequate sanitation and housing problem [10].

Sodium stigbogluconate (SSG) is the standardized and anti-monial drugs utilized for the treatment of VL in the United States and it is classified as an investigated new drug [11]. The recommended dose is 20 mg/kg per day for 20 days for cutaneous disease and 28 days for visceral or mucosal disease [12]. According to WHO report in 2010, the ideal therapeutic option for VL In Eastern part of Africa is the combined drugs types (sodium stigbo gluconate and paromomycin), which decrease the risk of relapse in PKDL(post kala-azar dermal leishmaniasis) and VL. It acts as a better curative therapeutic option for VL unless there is relapse condition [13]. Although its adverse effects have been increasingly reported, treatment with pentavalent antimonial agents has been regarded as safe for leishmaniasis. Pentavalent antimony can cause a wide range of adverse effects, including serious cardiotoxicity, hepatotoxicity, and acute pancreatitis. It may also lead to the permanent discontinuation of the treatment [14]. Acute pancreatitis (AP) is defined as an acute inflammatory condition of the pancreas with raised pancreatic enzymes levels in the blood and urine. In viseral leshimaniasis, the pathogenic leshimania parasite induce a decresea in the level of Th1 cells, which in turn leads to immune dysfunction and elevation of pro-inflammatory cytokines. The disease activity of VL is related with increased levels of pro-inflammatory cytokines, including inteleukin-6 (IL-6), IL8, and tumor necrosisTNF-α [15]. Hyperamylasemia and hyperlipasemia are observed in 98% of VL cases and nearly half of these patients were symptomatic [14]. The frequency of acute pancreatitis and hyperamylasemia complications are associated with antimonial therapy to be 20% and 40%, respectively [4]. The anti-parasitic treatment may be associated with abnormality in immune response and disease activity [16]. Even though paramomycin has toxicity, it act as a treatment option as the first line therapy become resistance to leshimania parasites. In Ethiopia, the other combined treatment (miltefosine and amphotericin B) reported by researchers as effective drugs for Viseral leshimaniasis patient infected with HIV [17]. The level of liver enzymes, including AST and ALT showed elevation in leshimaniasis patients with continuous treatment of Sodium stibogluconate [18]. VL patients showed the complication of splenomegaly and hepatomegally, which is associated with the disease [19].

Although various investigations were done on the prevalence of VL, no adequate comprehensive research were conducted in Ethiopia before this study. Majority of the studies focused on eipidmology and prevalence of the disease. They didn’t assess the biochemical parameters (serum amylase and lipase) regard to the VL and drug-induced clinical complications. In addition, there is no well-defined studies done regarding effect of SSG/paromomycin combined treatment on the normal functions of pancreas. Therefore, our study emphasize on the investigation of the effect of VL and combined treatment on the enzymes. The finding of this study may generate baseline information for clinicians and patients during the management of subsequent treatment. This research also provides baseline information that can be used as a reference for further studies on this area. It is also as an input for policy-makers of VL.

## Methods and materials

### Study area

Study was conducted at the University of Gondar Comprehensive Specialized Hospital Leishmaniasis Research and Treatment Center, with 24 beds capacity and its own laboratory, was built to reduce the disease burden. The hospital was established in 1954 and it is located in the central Gondar administrative zone, Amhara National Regional State, which is far about 750 km Northwest of Addis Ababa (the capital city of Ethiopia). Currently, Gondar town has one Comprehensive Specialized Hospital and eight government Health Centers. It serves approximately 5 million people and plays an important role in teaching students within the medical field more than 60 years. There are three leishmaniasis treatment centers in north west Ethiopia (Adiszemen, Metema and Abdurafi) and University of Gondar Comprehensive Specialized Hospital Leishmaniasis Treatment and Research Center is the only treatment and research center.

### Study design and period

An Institutional (hospital) based comparative cross-sectional study was conducted. The study was conducted at the Leishmaniasis Research and Treatment Center of University of Gondar Comprehensive Specialized Hospital from February to September 2020. The hospital is located in central Gondar administrative zone, North West Ethiopia.

### Source and study population

All lesishmaniasis patients who attended at Leishmaniasis Research and Treatment Center of University of Gondar Comprehensive Specialized Hospital were a source population for this study. On the other hand, VL patients who were eligible for the treatment of sodium stibogluconate and/or paromomycin drugs, at Leishmaniasis Treatment and Research Center University of Gondar Comprehensive Specialized Hospital were a study population during the study period.

### Eligibility criteria for patients

All voluntarily leishmaniasis patientseligibile for combined sodium stibogluconate with paromomycin drugs at the University of Gondar Comprehensive Specialized Hospital were included in this study. On the other hand, patients with alchol drinker and smoking habit and mental health problems, medication history and hearing impairment were excluded from this study.

### Sample size, sampling procedure and techniques

Many studies are based on relatively small sample size due to practical constraints such as time and subject availability, which often limits sample size [20]. Examining the sample size of other comparable studies carried out internationally assessing serum amylase and lipase sample size varies from 20 to 100 in these studies [4, 18, 21–23]. However, by using power analysis and sample size calculation by taking odds ratio value 2, the study participants were 100. Simple random sampling technique were used in the study period. First, take comprehensive patient history at day 0 which include abdominal symptom as a baseline information and 5 ml blood sample was collected at day 0 and 17 and the sample serum was taken to the lab to measure the required analytes) at day 0 and at 17. Laboratory results were checked for completeness on a daily basis by the immediate supervisor and the principal investigator. Before the data collection, training was given for data collector. The completed laboratory result was rechecked repeatedly by the principal investigator to maintain the quality of data.

### Data analysis process

The data was cleared, entered by EpiData version 3.1software and transferred to STATA version 14.1 SE. Simple descriptive statistics were used to analyze socio-demographic and clinical characteristics of the study subjects. Paired t-test was used to compare serum amylase and lipase level before and after treatment. Multivariate analysis was done to analyze the association between serum amylase and lipase with associated socio-demographic risk factors. The adjusted odd ratio was calculated for the multivariate analysis to assess risk factors for VL patients. All independent variables were checked by bivariate regression and p<0.25 was goes to multivariate analysis, p-value <0.05 at 95% confidence level was considered as statistically significant.

### Study variables

Serum amylase and lipase was the dependent variables whereas age, Sex, Marital status, educational, Occupational status, Religion, anthropometric measurement (BMI) and other demographic factors were served as the independent variable during the study. In addition, baseline serum amylase and lipase level was an independent variables.

### Ethics approval and informed consent

Ethical approval was obtained from the research and ethics committee of the Department of Biochemistry, School of Medicine, University of Gondar, Ethiopia. Department of ethics and research committee decided and approved the protocol with reference number, Ref. No 1944/03/2020.

## Results

### Socio-demographic characteristics of the study participants

In this study, a total of 100 VL patients who were under SSG and or paromomycin treatment at University of Gondar Comprehensive Specialized Hospital were selected during the study period. The Mean ± SD of ages among patients was 26.4 ± 2.2. Out of total 100 study participants, 33%, 48% and 19% of them were found under the age category of 18–24, 24–30 and 31–35 respectively. In this study,60 (60%), 35 (35%) and 5 (5%) study participants were single, married and divorced respectively. All the study participants were male. Concerning educational status, 58 (58%) and 12 (12%) patients completed elementary and secondary school respectively. Additionally, 30 (30%) of them were illiterate. Regarding residence area, 73% and 27% of them were from rural and urban areas respectively. From the total study participants, 60 (60%) were laborers, 29 (29%) farmers, 10 (10%) merchants and 1 (1%) were government employee. Majority of the participants were orthodox Christian (78%) and 22% were Muslims (as shown in Table 1).

**Table 1:** Socio-demographic characteristics of study participants.

Independent variables	Category	Total	
		Frequency (n)	Percentage (%)
Age (years)	18–24	33	33%
	25–30	48	48%
	31–35	19	19%
Marital status	Single	60	60%
	Married	35	35%
	Divorced	5	5%
	Widowed	0	0
Residence	Rural	73	73%
	Urban	27	27%
Occupational status	Laborer	60	60%
	Merchant	10	10%
	Government employee	1	1%
	Farmer	29	29%
Educational status	Illiterate	30	30%
	Primary school	58	58%
	Secondary school	12	12%
	College and university	0	0
Religious belief	Orthodox Christian	78	78%
	Muslim	22	22%
	Others	0	0

### Comparison of serum pancreatic amylase level before and after treatment in leishmanisis patients

**Fig 1:**
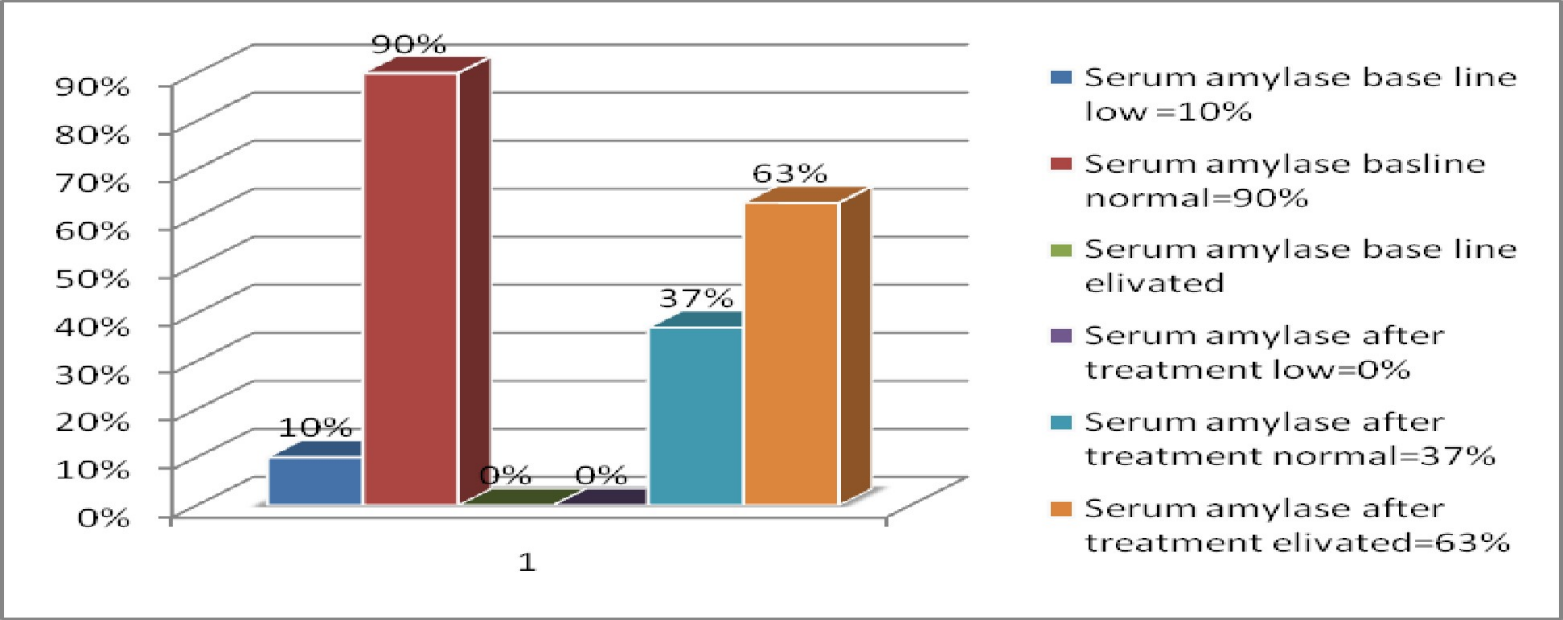


In this study, all of patients were confirmed VL cases through diagnosis of laboratory test (rk 39, splenic aspiration). In addition, they were under anti-leishmaniasis therapy with combined drugs of SSG and paromomycin. The baseline serum amylase level was measured before starting those drugs. The level of serum amylase and lipase were measured using DXC Beckman Coulter chemistry analyzer. According to this chemistry analyzer, the normal value of serum pancreatic amylase was in between 25-125U/L. In this study, majority of patient’s base line amylase value was normal (90%). In addition to this, 10% of them had lower value but there was no elevated serum amylase level at base line. However, majority of the result of serum amylase level at TOC were above the normal range. Thus, 63% of them had elevated serum amylase level (>125U/L), 37% of them had normal values (25–125 U/L) and no results were found below the normal level (as shown in Fig 1).

### Evaluation of serum amylase level before and after treatment using two tailed (paired) t-test statistics analysis

**Fig 2:**
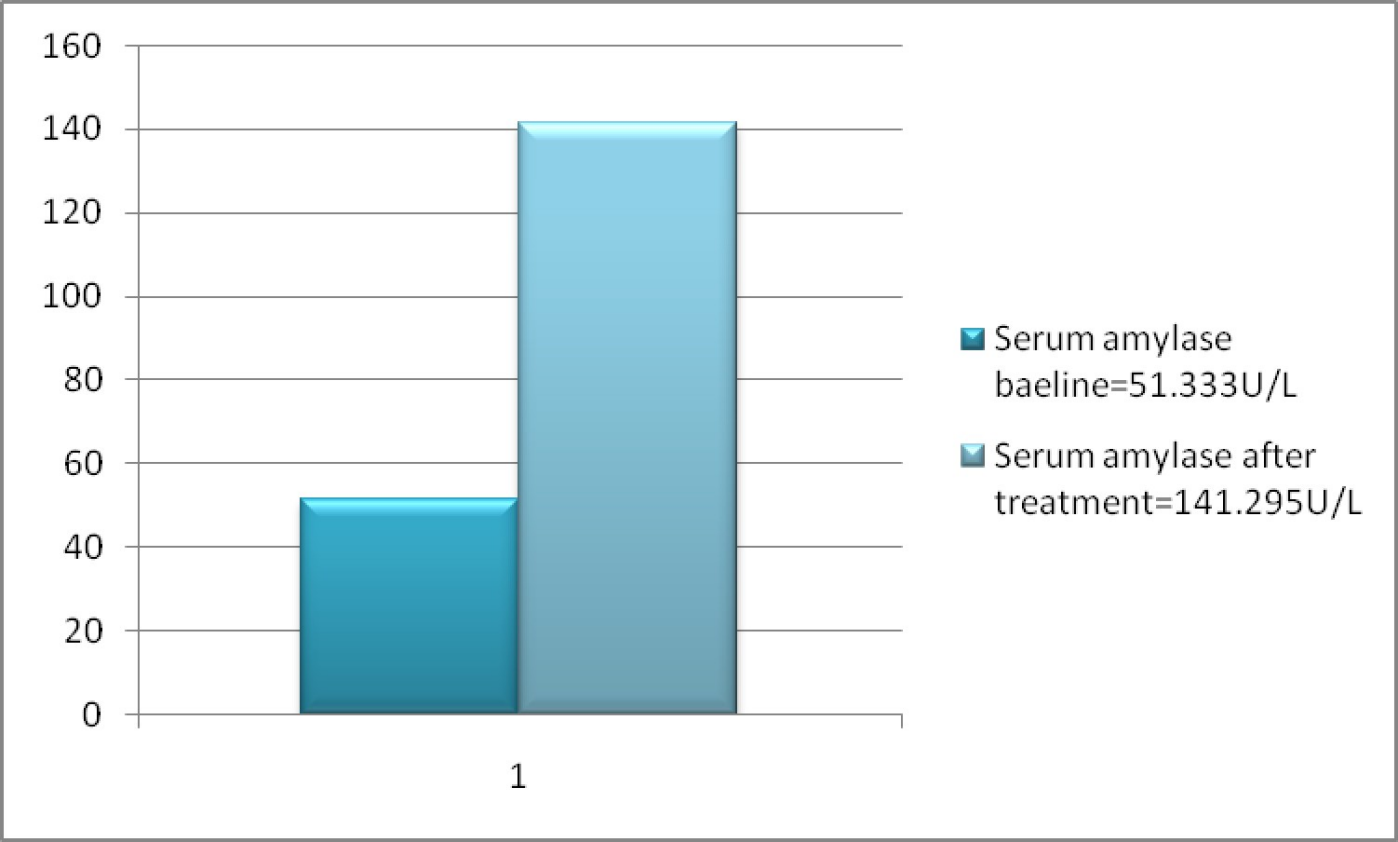


The level of serum amylase before and after treatment was analyzed using two tailed t-test statistics. Consequently, the value of amylase after treatment had a highly significant difference as compare its level before treatment(P< 0.001). The mean serum pancreatic amylase after treatment was higher than the mean serum pancreatic amylase before treatment (as shown in Table 2 and Fig 2)

**Table 2:** Comparison of serum amylase before and after treatment using paired t-test.

Variables	Observations	Mean	SD	95% CI		P-value
				Lower	Upper	
Baseline amylase(U/L)	100	51.33	19.47	47.49,	55.29	
Amylase at TOC (U/L)	100	141.29	39.07	133.54,	149.05	0.00*

### Correlation analysis of serum amylase level at TOC with age and base line amylase

In this study, pearson correlation analysis was made between pancreatic amylase and demographic characteristics (age). There was a statistically significant and positive association between serum pancreatic amylase at TOC with age (r = 0.23 and p value<0.05) and with baseline serum pancreatic amylase (r = 0.19 and p = 0.05). Pearson`s correlation of the serum amylase at TOC with age and base line serum amylase (as shown in Table 3).

**Table 3:** Pearson correlation of amylase at treatment of cure with base line amylase and age.

Variables	Amylase after treatment	
	r	P-value
**Age**	0.23	**0.023** *
**Amylase before treatment**	0.19	0.05

* P-values is significant at <0.05*.

### Comparison of serum pancreatic lipase before and after treatment in leishemaniasis patients

From the total enrolled study participants, the result of baseline serum lipase level showed that 85% of study participants had normal value (11–82 IU/L). During baseline analysis, 12% and 3% of study subjects had lower (<11 IU/L) and higher value (>82 IU/L) respectively. After treatment, serum lipase level was elevated among 78% of study subjects (>82 IU/L). On the other hand, 22% of them had normal value (11–82 IU/L) and none of them had lower serum lipase level.

### Evaluation of serum lipase level before and after treatment by using paired t-test

The mean serum pancreatic lipase after treatment showed statistically significant elevation as compared to its value before treatment (p<0.001). In two tailed paired t-test statistics, the serum lipase at TOC was 123.94, serum lipase at base line was 37.16 (p-value of 0.000) (as shown in Table 4 and Fig 4).

**Table 4:** Paired t-test comparing serum pancreatic lipase before and after treatment.

Variables	Observation	Mean	SD	95% CI		p-value
				Lower	Upper	
Baseline lipase(IU/L)	100	37.1593	20.811	33.02	41.2	
Lipase TOC(IU/L)	100	123.9422	47.07	114.60	133.28	0.00*

(command in statattestLipase_Baseline = = Lipase_End_Treatment), TOC = test of cure, obs = observation, SD = standard deviation, CI = confidence interval and p-value <0.05 was significant*).

### Correlation analysis of serum lipase at TOC with age and base line lipase

There was statistically significant positive correlation between serum lipase at TOC with age (r = 0.2022 and p-value = 0.0429*) and with base line serum lipase (r = 0.24 and p-value = 0.01). Pearson correlation between serum lipase at TOC with age and base line lipase (as shown in Table 5).

**Table 5:** Pearson correlation between serum pancreatic lipase at treatment of cure with base line lipase and age.

Variable	Lipase after treatment	
	R	P-value
**Lipase before treatment**	0.24	0.01*
**Age**	0.2022	0.0429*

(* P-values is significant at <0.05.*r is correlation coefficient).

### Multiple linear regression analysis of serum amylase after treatment in patients taking sodium stibogluconate and or paromomycin drugs

All the assumptions, including linearity, independence, normality, equality of variance, and multicollinearity were checked by binary linear regression to perform multilinear linear regression analysis. Multivariate analysis was done for study variables, including age, educational status, religious, and baseline amylase. It showed that serum amylase after treatment had a significant association with age (r = 2.25, p-value = 0.022) and baseline amylase (r = 0.366, p-value = 0.05) level. It indicates the presence of a one year increase in age increases serum amylase after treatment by 2.25. A one unit increase of base line serum amylase increase serum amylase after treatment by 0.367 keeping other variables constant. Multivariate analysis of serum amylase with its baseline value and age expressed (as shown in Table 6).

**Table 6:** Multiple linear regression between serum amylase at test of cure with different factor variables.

Characteristics	Crude coefficient 95CI of β	p-value	Adjusted coefficient 95CI of β	P-value
**Age**	2.2	0.024*	2.25	0.022*
**Amylase base line**	0.38	0.05*	0.366	0.05*
**Educational status**	-7.89	0.21	-2.75	0.68
**Religious belief**	-11.0	0.245	-13.66	0.179

(*p-value was significant at 0.05* βo coefficient*).

### Multiple linear regression analysis of serum lipase after treatment in a patient taking sodium stibogluconate/paromomycin combined drugs

All independent variables were checked by bivariate regression (p<0.25) to perform multivariate analysis. During multivariate analysis, age (r = 2.11, p = 0.05) and lipase line (r = 0.5, p = 0.025) were found significantly associated with serum lipase after treatment. It indicates the presence of a one year increase in age increases serum lipase at TOC by 2.11 and a one unit increase in base line serum lipase increase serum lipase at TOC by 0.5. Multiple linear regression of Serum lipase after treatment with different variables in a patient taking sodium stibogluconate/paromomycin drugs (as shown in Table 7).

**Table 7:** Multiple linear regression between serum pancreatic lipase with different factor variables.

Characteristics	Crude coefficient 95CI of β	p-value	Adjusted coefficient 95CI of β	P-value
**Age**	2.372	0.043*	2.11	0.05*
**Educational status**	-8.824	0.245*	-5.53	0.454
**Lipase base line**	0.55	0.015*	0.50	0.025*

(*p-value was significant at 0.05* βo coefficient*).

## Discussion

Leishmaniasis is a protozoal disease caused by an obligate intra-macrophage protozoan parasite, which is endemic in large areas of the tropics, subtropics and the Mediterranean basin. It consists of four main clinical syndromes, such as cutaneous leishmaniasis, muco-cutaneous leishmaniasis, VL, and post-kala-azar dermal leishmaniasis (PKDL). VL is a systemic fatal disease if it is untreated and it is caused by the *Leishmaniadonovani c*omplex. Low income countries, including East African countries and the Indian sub-continents are particularly affected by the disease [24]. In low land and arid areas of Ethiopia, it becomes a serious public health burden and it has high prevalence with an estimated annual incidence of more than 4,000 cases [25]. Currently, the treatment choice of VL in East Africa for immune-competent individuals are sodium stibogluconate and paromomycin combined drugs. The study was conducted at University of Gondar Comprehensive Specialized Hospital, Leishimaniasis Treatment and Research Center to assess serum pancreatic amylase and lipase before and after treatment. In this study, we evaluated serum levels of amylase and lipase among study participants undergoing VL treatment with the same therapeutic regimen. All patients were immune-competent and none of them had risk factors, including medication, HIV/AIDS, and trauma, which may induce acute pancreatitis [26]. We assessed the effect of these drugs on the pancreas by evaluating pancreatic enzymes. We investigated the level of serum amylase and lipase before and after treatment among VL patients. The study was conducted among 100 study participants. All the study participants were male with mean ages 26±2.2 years (range from 18–35). There are other similar conducted studies, which involve only males as study participants [23, 27]. Majority of the study subjects were single(60%), completed elementary school(58%), laborer(60%), from rural area (73%) and orthodox Christian (78%) (Table 1). We calculated the BMI of the study participants using their weight and height. The mean ±SD was found to be 16.6±1.1kg/m2. There were no similar studies reported yet, which assessed BMI of the patients previously. Treatment with pentavalent antimonial agents has been regarded as safe for leishmaniasis even if it has increased adverse effects. It causes a wide range of adverse effects, including cardiotoxicity, hepatotoxicity and acute pancreatitis. In the previous study, acute pancreatitis by anti-monial therapy, was reported as one of the adverse effect [28–30]. Similarly, Delgado and his co-workers also showed the frequency of acute pancreatitis and hyperamylasemia as complication associated with antimonial therapy was 20% [31] and 40% [28], respectively. Nearly half of these patients were symptomatic.

**Fig 3:**
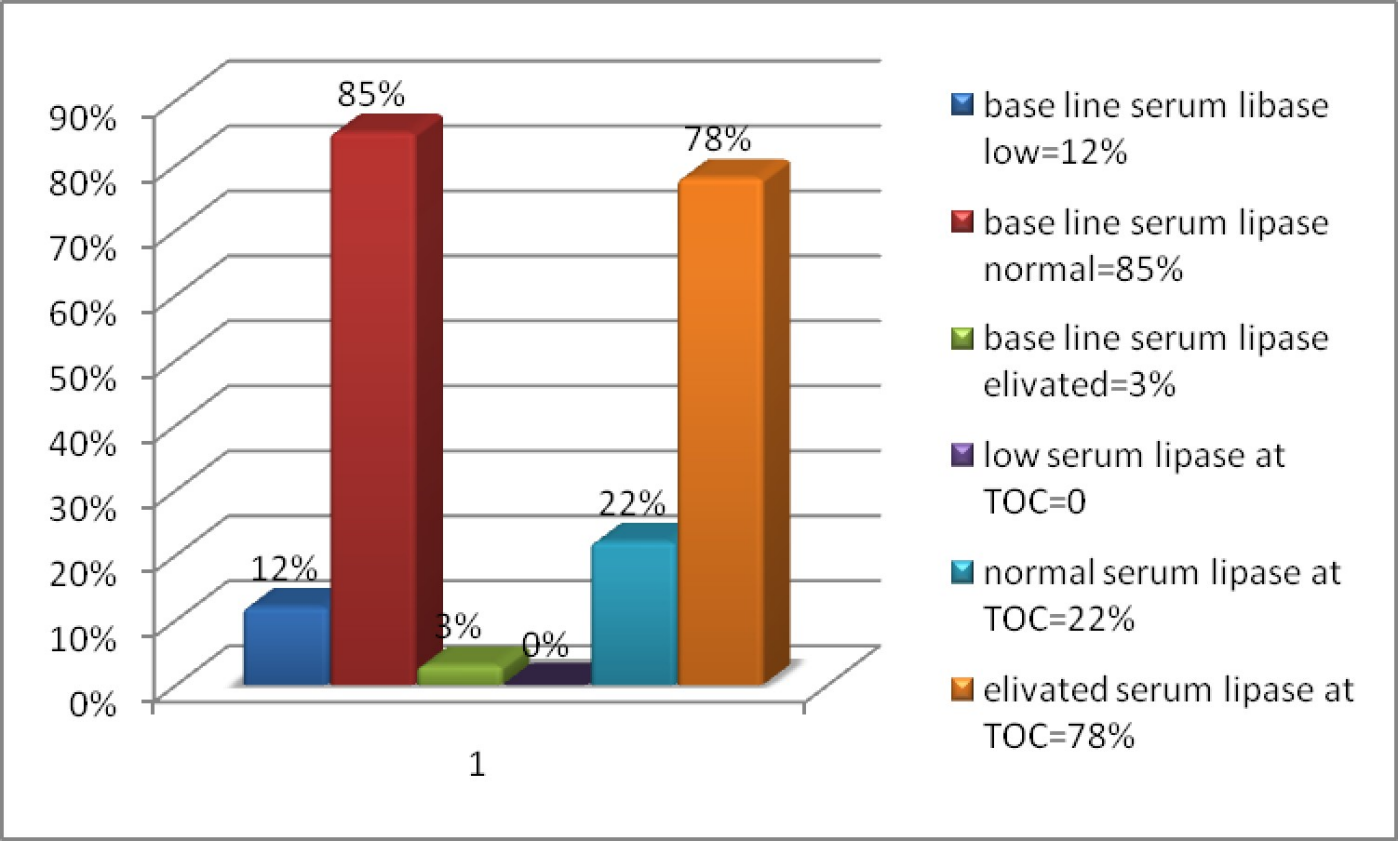


In this study, we measured serum levels of amylase and lipase among100 patients, which was diagnosed as VL. From all study participants, at the end of treatment 37% (37) of them has normal and 63% (63) had elevated serum amylase level as compared to normal base line serum amylase level (Fig 1). Consequently, the value of amylase after treatment had a highly significant difference as compared to its level before treatment. In this study, 22 (22%) patients had normal serum lipase levels, whereas 78(78%) VL patients showed elevated serum lipase level after treatment. On the other hand, 12 (12%), 85 (85%) and 3 (3%) patients had low, normal and elevated baseline serum lipase level respectively (Fig 3). Thus, the result showed that there was a rough difference of serum amylase and lipase between before and after treatment. Some of the study participants had a characteristic manifestation of abdominal symptom. This result disagreed to the study conducted by Lyra et al. [4, 18], which showed fifty percent presented some gastrointestinal manifestation compatible with pancreatitis: anorexia (33.3%), nausea (29.1%), vomiting (15.3%) and abdominal pain (18.0%). From 36 patients with gastrointestinal complaints, 47.2% had increased serum lipase or amylase [18]. This deviation from our study might be due to different age group and geographical difference.

**Fig 4:**
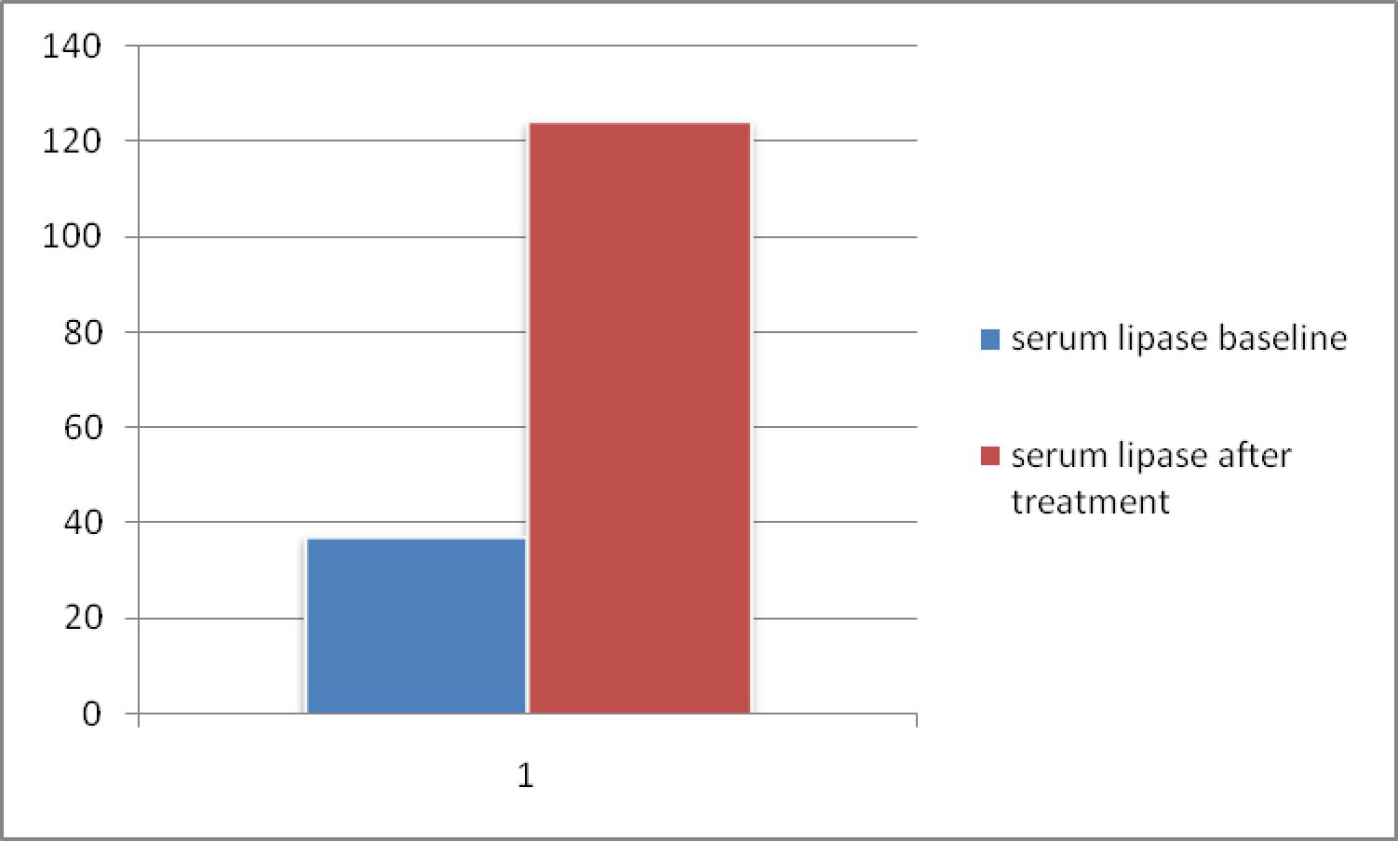


This study was also inconsistent to the studies conducted by Aronson et al. [23], which reported that 86% of VL patients had elevated serum amylase and 100% elevated serum lipase with 82.7% abdominal symptoms and 81.48% nausea and vomiting. The difference to this might be there were no exclusion criteria in which a factor that induce acute pancreatitis to that particular studies and due to different age group. However, our study excludes the factors that induces acute pancreatitis. A paired or dependent t-test was done and it showed a statistically significant difference among serum pancreatic amylase and lipase before and after treatment with a p-value of <0.001. There fore, serum amylase at TOC was 141.29±39.07 U/L as compared to (51.33 ±19.48 U/L) base line serum amylase (Fig 2 and Table 2). Similarly, the mean ± SD of serum lipase at TOC and before treatment was 123.9±47.07 U/L and 37.1593±20.81 U/L respectively. Thus, there was a statistically high significant elevation of lipase at test of cure as compared to the baseline level (p<0.001) (Fig 4 and Table 4). Consequently, there was a statistically significant difference between serum amylase and lipase before and after treatment. In line to our study, the study conducted by Gasser et al. showed that 63(63%) study participants had elevated serum amylase at TOC, whereas 78 (78%) patients had elevated serum lipase level at TOC. Additionally, the study conducted earlier by Aronson et al. [23] revealed that, 86% of study subjects had elevated serum amylase. Aronson and his co-workers also notified that all of the participants had elevated serum lipase level. In line to this previous study, the result of our study confirmed the presence of elevated serum lipase level. The study conducted by Lyra et al., didn’t show the presence of significant association of hyperamylasemia and hyperlipasemia with any socio-demographic studied variables [18]. It disagreed with our study, since in our study the correlation between serum amylase at TOC showed that there was a statistically significant and positive correlation with age (r = 0.2262 and p value<0.05) and serum lipase at TOC to age (r = 0.2022 and p-value<0.05). Our study also disagreed with the study conducted by Lyra et al., in which serum amylase and lipase at TOC were not correlated to factor variables [18]. The difference may be due to geographical and age distribution difference. On the contrary, there was no either positive or negative significant relation among height, weight and BMI of both serum amylase and lipase at TOC. As far as our search is concerned there was no similar studies done yet to correlate amylase and lipase from anthropometric measurements (Height, Weight and BMI).

There was also a statistically significant positive correlation between serum amylase at TOC with base line serum amylase (r = 0.19, and p = 0.05). Similarly, serum lipase at TOC was also statistically significant and positive correlation with base line serum lipase with (r = 0.2432, p = 0.0148)), so these showed that increased of the base line serum amylase and lipase would increase their irrespective serum amylase and lipase at TOC respectively. There was no previous finding which showed correlation analysis of base line amylase with amylase at TOC and base line lipase with lipase at TOC. In this study, an increment of amylase at TOC was accompanied by serum lipase at TOC, and it was supported by the study conducted by Lyra et al. and his co-workers, which showed ahperamylasemia was always accompanied by hyperlipasemia.

On the other hand, multivariate analysis was done to evaluate the association between serum amylase and lipase level with socio-demographic characteristics. In this study, Significant variables to serum amylase at TOC was significantly associated with age with B_0 =_ 2.2, p-value = 0.22 and baseline serum amylase constant, B_0 =_ 0.78, p-value = 0.05 (Table 3). It means a one year increase in age increases serum amylase at TOC by 2.2 units by keeping other variables as a constant. In addition to this, a one unit increase of base line serum amylase increase serum amylase at TOC by 0.78 units (Table 5). This study disagreed with the earlier studies of Lyra et al., in which there were no correlation between amylase at TOC to age and baseline serum amylase. Concerning to serum lipase at TOC it was significantly associated with age(B_0 =_ 2.1138, p = 0.05) and base line serum lipase(B_0_ = 0.5, p = 0.025). This is also interpreted as there was 2.1138 unit increase of serum lipase at TOC with a one year increase of age. Similarly, serum lipase at TOC was increased by 0.5 in a one unit increase of baseline serum lipase (Tables 6 and 7).

## Conclusion

The result of this study showed that the prevalence of high serum amylase and lipase at TOC was higher and there was an increase in mean serum amylase and lipase after a patient taking sodium stibogluconate and or paromomycin drugs. The significant elevation in serum amylase and lipase level may be due to the effect of those combined drugs rather than VL associated complication. A high prevalence of drug induced pancreatitis, which has been detected in elevated level of these serum enzymes. Additionally, there were a statistically significant association between amylase at TOC with age and amylase base line. Similarly, lipase at TOC showed a statistically significant correlation with age and baseline lipase value. Serum lipase and amylase elevated as the age of the patient increased, which showed an additional exposure risk factor for abnormality of these biochemical parameters. Consequently, the elevated result of these biochemical profiles mainly associated with drug induced adverse effects and associated risk factors.

## List of abbreviations

CL: Cutaneous Leishmaniasis
PKDL: post ka-alazar dermal leishimaniasis
PM: Paramomycin
SSG: Sodium StigboGluconate
TOC: test of cure
VL: Visceral Leishmaniasis
MCL: mucocutaneous Leishmaniasis
